# Myofibrillar protein synthesis following ingestion of soy protein isolate at rest and after resistance exercise in elderly men

**DOI:** 10.1186/1743-7075-9-57

**Published:** 2012-06-14

**Authors:** Yifan Yang, Tyler A Churchward-Venne, Nicholas A Burd, Leigh Breen, Mark A Tarnopolsky, Stuart M Phillips

**Affiliations:** 1 Exercise Metabolism Research Group, Department of Kinesiology, McMaster University, 1280 Main St. West, Hamilton, ON, Canada, L8S 4K1; 2 Michael G. DeGroote School of Medicine, Pediatrics and Neurology, McMaster University, Hamilton, ON, Canada

**Keywords:** Soy protein, Myofibrillar protein synthesis, Elderly, Resistance exercise

## Abstract

**Background:**

Increased amino acid availability stimulates muscle protein synthesis, however, aged muscle appears less responsive to the anabolic effects of amino acids when compared to the young. We aimed to compare changes in myofibrillar protein synthesis (MPS) in elderly men at rest and after resistance exercise following ingestion of different doses of soy protein and compare the responses to those we previously observed with ingestion of whey protein isolate.

**Methods:**

Thirty elderly men (age 71 ± 5 y) completed a bout of unilateral knee-extensor resistance exercise prior to ingesting no protein (0 g), or either 20 g or 40 g of soy protein isolate (0, S20, and S40 respectively). We compared these responses to previous responses from similar aged men who had ingested 20 g and 40 g of whey protein isolate (W20 and W40). A primed constant infusion of L-[1-^13^ C]leucine and L-[*ring*-^13^ C_6_]phenylalanine and skeletal muscle biopsies were used to measure whole-body leucine oxidation and MPS over 4 h post-protein consumption in both exercised and non-exercised legs.

**Results:**

Whole-body leucine oxidation increased with protein ingestion and was significantly greater for S20 vs. W20 (*P* = 0.003). Rates of MPS for S20 were less than W20 (*P* = 0.02) and not different from 0 g (*P* = 0.41) in both exercised and non-exercised leg muscles. For S40, MPS was also reduced compared with W40 under both rested and post-exercise conditions (both *P* < 0.005); however S40 increased MPS greater than 0 g under post-exercise conditions (*P* = 0.04).

**Conclusions:**

The relationship between protein intake and MPS is both dose and protein source-dependent, with isolated soy showing a reduced ability, as compared to isolated whey protein, to stimulate MPS under both rested and post-exercise conditions. These differences may relate to the lower postprandial leucinemia and greater rates of amino acid oxidation following ingestion of soy versus whey protein.

## Introduction

Ageing is associated with sarcopenia [1] that ultimately results from an imbalance between rates of muscle protein synthesis and breakdown. Both physical activity and nutrient availability represent potent anabolic stimuli for adult muscle, however, the ability of elderly muscle to mount a robust increase in myofibrillar protein synthesis (MPS) in response to amino acids [2,3] and resistance exercise [4] is attenuated compared to that seen in the young; a phenomenon termed ‘anabolic resistance’ [2]. Previous studies have shown that both protein dose [2,5,6] and source (i.e., plant vs. animal) [7-11] are important in determining the postprandial response of MPS, which may be of particular relevance to the elderly. For example, we have recently demonstrated greater increases in post-exercise MPS in the elderly following bolus ingestion of 40 g vs. 20 g of whey protein [6]; a finding in contrast to our data from young adults who show a maximal MPS response with 20 g protein and no further increase with 40 g [5]. Thus, it appears that higher doses of protein [6,12], and/or leucine [13,14] to promote a greater aminoacidemia or leucinemia [7] are required by the elderly to maximize the response of MPS to protein ingestion.

The mechanisms underpinning the differential capacity of proteins from different sources to support increased rates of protein synthesis are not fully understood [15]. Whey protein [7,9,10] and bovine milk [8] (~20% whey protein) appear to stimulate greater rates of muscle protein synthesis than do proteins such as micellar casein or soy both at rest and following resistance exercise. This is somewhat counter-intuitive given that soy, whey, and casein are all defined as high quality proteins based on their protein digestibility corrected amino acid scores (PDCAAS; for review see [16]). However, the digestion kinetics of these proteins is markedly different, and protein digestibility has been established as an important factor regulating whole-body protein synthesis and breakdown [17,18]. Both whey [18] and soy [19] are acid soluble, a characteristic that facilitates rapid digestion and results in a large but transient increase in aminoacidemia. These so-called ‘fast’ proteins induce a rapid aminoacidemia and appear to support greater increases in MPS. On the other hand ‘slow’ proteins, such as micellar casein (which clots in the acidic pH of the stomach) is slowly digested and induces a more moderate but sustained aminoacidemia than whey [7,10].

Knowledge of the capacity of proteins from different sources to stimulate MPS in the elderly is warranted in view of the importance of preserving skeletal muscle mass in ageing. Therefore, the aim of the current study was to examine the effects of different doses (20 g and 40 g) of soy protein isolate on MPS at rest and following the potent anabolic condition of resistance exercise in elderly men, and compare these findings to our previous work examining the effects of graded intakes of whey protein isolate on MPS in the elderly [6].

## Methods

### Participants

Thirty elderly men (age 71 ± 5 y, BMI 26 ± 3 kg·m^2^) were recruited to participate in the study and were randomly assigned to one of three treatment groups that were counterbalanced for body mass, age, and self-reported physical activity levels. Participants were light-to-moderately active, non-smokers, non-diabetic, and considered generally healthy based on responses to a routine health screening questionnaire. Participants taking medications controlling blood pressure were allowed into the study. The characteristics of the whey protein treatment groups (W20, W40) have been reported previously [6], but are shown again in Table 1 along with the control (0 g) and soy protein treatment groups (S20, S40) for reader comparison. Participants were informed of the purpose of the study, the associated experimental procedures, and any potential risks prior to providing written consent. The study was approved by the local Health Sciences Research Ethics Board at McMaster University and conformed to standards for the use of human participants in research as outlined in the 5th Declaration of Helsinki and with current Canadian funding agency guidelines for use of human participants in research [20].

**Table 1:** Participant characteristics

**Parameter**	**0 g (n = 10)**	**W20 (n = 10)**	**W40 (n = 10)**	**S20 (n = 10)**	**S40 (n = 10)**
Age (y)	71 ± 5	72 ± 5	70 ± 4	72 ± 6	70 ± 5
Total body mass (kg)	78 ± 13	81 ± 9	81 ± 12	78 ± 11	77 ± 9
Fat free mass (kg)	55 ± 9	57 ± 6	56 ± 9	55 ± 6	53 ± 6
% Body fat	26 ± 5	26 ± 4	27 ± 8	25 ± 5	26 ± 6
BMD (g·cm^2^)	1.19 ± 0.11	1.20 ± 0.11	1.23 ± 0.11	1.25 ± 0.08	1.28 ± 0.11
Height (m)	1.73 ± 0.06	1.76 ± 0.06	1.75 ± 0.09	1.71 ± 0.09	1.74 ± 0.06
BMI (kg·m^2^)	25.9 ± 3.4	26.2 ± 2.8	26.0 ± 2.2	26.6 ± 3.7	25.5 ± 2.7
Systolic BP (mmHg)	136 ± 15	134 ± 19	129 ± 14	124 ± 13	127 ± 12
Diastolic BP (mmHg)	80 ± 10	72 ± 8	78 ± 5	73 ± 9	72 ± 8
Total SPBB score	11.7 ± 0.5	11.3 ± 0.7	11.6 ± 0.7	11.8 ± 0.4	11.4 ± 1.0

Values are means ± SD. BP: Blood pressure, SPBB: Short Physical Performance Battery. Total SPBB score calculated as the sum of walk test, chair stand and balance tests.

### General design

The different groups of older men ingested 20 g or 40 g of soy protein isolate in beverage form after performing an acute bout of unilateral knee-extensor resistance exercise. Employing a unilateral exercise model allowed us to examine the effect of protein intake alone, and the interaction of exercise and protein intake within the same individual.

### Preliminary assessments

One week prior to the experimental infusion trial, body mass and composition were assessed via a dual energy X-ray absorptiometry (DXA) scan (Table 1). Physical performance was assessed using the Short Physical Performance Battery (SPPB) [21], consisting of a 3-4 m walk test, chair stand, and balance test (Total SPPB score presented in Table 1). Health parameters were also assessed and included systolic and diastolic blood pressure, resting heart rate, and the following blood parameters: fasting glucose, triglycerides, total cholesterol, high density lipoprotein (HDL), low density lipoprotein and ratio of total cholesterol to HDL. At least one week prior to the experimental infusion trial, participants underwent a strength test to determine their unilateral 10 repetition maximum (RM) on a standard knee extension machine as previously described [9].

### Dietary control

Participants were required to complete diet records prior to initiating the study to provide an estimate of habitual macronutrient intake as analyzed using a commercially available software program (Nutritionist V, First Data Bank, San Bruno, CA). Reference lists for portion size estimates were provided to the study participants, who were instructed to record all food or drink consumed in a diet log during a 3-day period (i.e., 2 weekdays and 1 weekend day; see Additional file 1: Table S1). Two days prior to the trial, participants were supplied with pre-packaged diets that provided a moderate protein intake (1.0 g·kg^-1^·d^-1^). Energy requirements for the controlled diets were estimated via the Harris-Benedict equation and were adjusted using an activity factor calculated for each individual subject based on their self reported physical activity. Body mass was monitored over the course of the controlled diet period to ensure participants were in energy balance. Participants were instructed to abstain from any strenuous exercise until after completion of the trial.

### Infusion protocol

Participants reported to the laboratory at ~0700 in a 10 h post-absorptive state. Upon arriving at the laboratory, a baseline breath sample was collected to measure ^13^CO_2_ enrichment via isotope ratio mass spectrometry (BreathMat Plus; Finnigan MAT GmbH, Bremen, Germany). A plastic catheter was then inserted into an antecubital vein and a baseline blood sample was collected before initiating a 0.9% saline drip to keep the catheter patent for repeated blood sampling during the infusion trial. After baseline breath and blood samples were taken, a bout of unilateral knee-extensor resistance exercise was performed on a guided-motion knee extension machine. The exercise bout involved 3 sets, using a pre-determined load based on each participant’s 10RM. Each set was completed within ~25 s with an interest rest interval of 2 min. Immediately following exercise, blood and breath samples were obtained and a second catheter was inserted into the contralateral antecubital vein to prime the bicarbonate pool with NaH^13^CO_2_ (2.35 μmol·kg). Thereafter, priming doses of [1-^13^ C] leucine (7.6 μmol·kg^-1^) and L-*ring-*^13^ C_6_ phenylalanine (2 μmol·kg^-1^; 99 atom percent; Cambridge Isotopes, Andover, MA) were introduced, before a continuous infusion of L-[1-^13^ C] leucine (7.6 μmol·kg^-1^·h^-1^) and L-*ring-*^13^ C_6_ phenylalanine was initiated (0.05 μmol·kg^-1^·min^-1^). Arterialized blood samples were obtained by wrapping the forearm in a heating blanket (45°C) for the duration of the infusion; a procedure we have found completely arterializes venous blood sampled from a hand vein. Blood samples were processed as previously described [5]. Immediately after post-exercise blood and breath samples had been obtained, participants consumed water (0 g) or a drink containing 20 g or 40 g of either whey or soy protein isolate (W20, W40, S20, S40) dissolved in 400 mL water. The whey protein was generously donated by PGP International (IWPI 9500, California, USA), while the soy protein was generously donated by the Solae Company (SUPRO 660-IP, St Louis, MO). The amino acid composition of both the whey and soy protein drinks is provided in Additional file 2: Table S2. On the basis of a leucine content of 10% in whey and 8% in soy, and a phenylalanine content of 3% in whey and 5% in soy protein, drinks were enriched to 8% with [1-^13^ C] leucine and 8% with ^13^ C_6_ phenylalanine to minimize disturbances in isotopic steady state; an approach that we have validated [22]. Complete drink consumption was considered *t* = 0 min and the isotopic infusion was continued until *t* = 240 min. During the remainder of the infusion, arterialized blood and breath samples were obtained to confirm steady state and measure leucine oxidation and MPS as previously described [5,8]. At the end of the infusion (*t* = 240 min) muscle biopsies were obtained (described below).

### Muscle biopsy sampling

Muscle biopsy samples were obtained from the *vastus lateralis* muscle from both exercise and non-exercised legs using a 5-mm Bergstrm needle (modified for manual suction), under 2% local anaesthesia by xylocaine. Muscle biopsies were freed from any visible blood, fat, and connective tissue and rapidly frozen in liquid nitrogen until further analysis.

### Blood analyses

Plasma L-*ring*^13^ C_6_ phenylalanine enrichments were determined as previously described [23]. Blood amino acid concentrations were analyzed by HPLC as previously described [24]. Plasma insulin was measured using a commercially available immunoassay kit (ALPCO Diagnostics, Salem, NH, USA) following the manufacturer instructions.

### Muscle analyses

Myofibrillar enriched protein fractions were isolated from ~30 mg of wet muscle as described previously [25]. Intracellular amino acids (IC) were isolated from a separate piece of wet muscle (~25 mg) as previously described [26].

### Calculations

The fractional synthetic rates (FSR) of myofibrillar proteins were calculated using the standard precursor-product method:

(1) $$\text{FSR}\left(\%\text{h}-1\right)={\text{E}}_{\text{p}2}-{\text{ E}}_{\text{p}1}/{\text{E}}_{\text{ic}}\text{x }1/t\text{x }100$$

where E_p2_ and E_p1_ are the protein bound enrichments from muscle biopsies at 240 min and baseline plasma proteins, respectively. The difference represents the change in bound protein enrichment between two time points; E_ic_ is the mean intracellular phenylalanine enrichment from the biopsies; and *t* is the tracer incorporation time. The utilization of ‘tracer naive’ subjects allowed us to use the pre-infusion blood sample (i.e., mixed plasma protein fraction) as a surrogate baseline enrichment of muscle protein; an approach we have previously validated [26] and that has been validated by others [27]. Previously, others have used a pre-infusion muscle biopsy and found equivalent rates of muscle protein synthesis and shown such an approach [28] to be valid. We have found baseline plasma enrichment to be equivalent to that of pre-infused muscle (unpublished results), indicating that there is little reason in using a pre-infusion biopsy over a blood sample for baseline enrichment.

Leucine oxidation was calculated as described in our previous publications [5,8] from the appearance of the ^13^ C-label in expired CO_2_ using the reciprocal pool model with fractional bicarbonate retention factors of 0.7 and 0.83 for fasted (0 g protein) and fed (S20 and S40) states, respectively [29]. The area under the leucine oxidation by time curve was calculated using GraphPad Prism 5 (San Diego, CA) as an estimate of total leucine oxidation [5,8].

### Statistical analyses

A 3-way ANOVA with both between (protein dose and protein source), and within (condition) subject factors was used. When a 3-way interaction was found (i.e. between protein dose, protein source, and condition (rest vs. exercise)) analyses of variance was used to examine individual time and dose effects and isolate significant pairwise differences by calculating critical differences and by comparisons of means accounting for differences in the means by time. Following observation of a significant *F* ratio by ANOVA, a Tukey’s honestly significantly different test, with adjustment for multiple comparisons, was used for post hoc analyses. Significance was set at *P* ≤ 0.05. All statistical analyses were performed using SPSS 17 for Windows.

## Results

### Participant characteristics

There were no between-group differences in age, body weight, body composition, SPBB or other subject characteristics (Table 1). Dietary intake for the 2 day run-in prior to the study was similar for all groups (Additional file 1: Table S1).

### Plasma insulin

**Figure 1:**
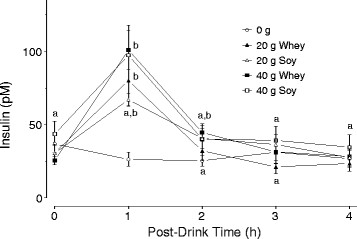
**Plasma insulin in the 0 g protein group, and in the whey (W20, W40) and soy (S20, S40) groups.** Means with different letters are significantly different from time 0 and from each other (*P* < 0.05). Data are means ± SD.

Plasma insulin concentration was similar for all groups at 0, 3 and 4 h post-drink. At 1 h post-drink, insulin concentration had increased by ~2.6- and 4-fold for W20 and W40, and ~2.2 fold for both S20 and S40 (Figure 1).

### Plasma amino acids

**Figure 2:**
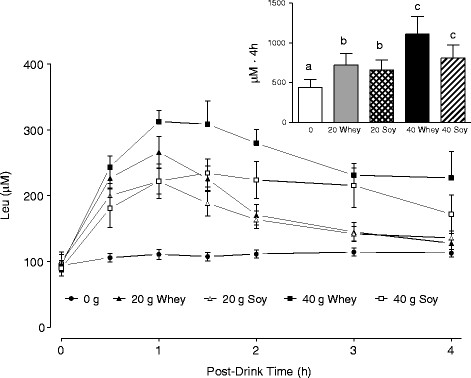
**Blood leucine in the 0 g protein group, and in the whey (W20, W40) and soy (S20, S40) groups (no statistical analysis is shown except on the area under the concentration-time curve, see inset).** Means with different letters are significantly different (*P* < 0.05). Data are means ± SD.

**Figure 3:**
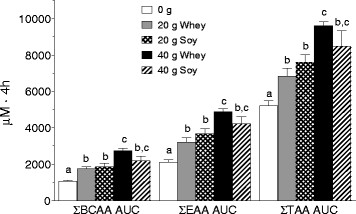
**Area under the blood amino acid concentration time curves (AUC) in the 0 g protein group, and in the whey (W20, W40) and soy (S20, S40) groups for summed total of branched-chain amino acids (BCAA), summed total of the essential amino acids (EAA, including His) and summed total of all amino acids (total AA, excluding Cys and Trp).** Means with different letters are significantly different (*P* < 0.05). Data are means ± SD.

Peak blood leucine concentration occurred between 1.0-1.5 h post-drink for S20, W20, and W40, but occurred at ~1.5-2.0 h for S40 (Figure 2). Higher peak amplitudes in blood leucinemia were achieved following whey as compared to soy protein regardless of dose (P < 0.05). Area under the curve (AUC) for leucine increased in a stepwise manner from 20 g to 40 g of protein with no difference between protein sources (Figure 2 Inset). Blood BCAA, EAA, and Total amino acids increased with protein ingestion (i.e., versus 0 g), however there were no differences between protein sources (Figure 3). While the AUC for leucine, BCAA, EAA, and Total amino acids was greater for W40 vs. W20, there were no significant differences in these amino acid concentrations between S40 vs. S20.

### Whole-body leucine oxidation

**Figure 4:**
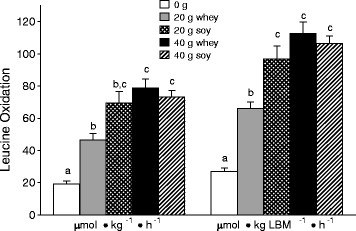
**Whole-body leucine oxidation at different protein doses expressed relative to total body weight (left) and to lean body mass (right) in the 0 g protein group, and in the whey (W20, W40) and soy (S20, S40) groups.** Means with different letters are significantly different (*P* < 0.05). Data are means ± SD.

Whole-body leucine oxidation AUC increased with protein intake. When expressed relative to lean body mass, the increase in whole body leucine oxidation for S20 was significantly greater than W20 (*P* = 0.002). There were no differences in leucine oxidation between S40 and W40 (Figure 4).

### Myofibrillar protein fractional synthetic rate (FSR)

**Figure 5:**
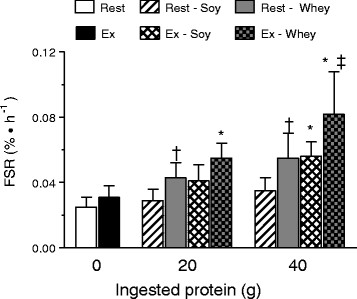
**Myofibrillar protein fractional synthetic rate (%·h**^**-1**^**) for whey and soy (20 g and 40 g) groups and a group who consumed no protein (0 g) at rest and following resistance exercise (Ex) as described.** There was a significant dose by condition by protein source interaction (*P* < 0.001). † Indicates a significant (*P* < 0.05) difference from the 0 g dose within the Rest condition; * indicates a significant (*P* < 0.05) difference from the 0 g dose within the Ex condition; and ‡ indicates a significant (*P* < 0.05) difference from the 20 g Ex condition within the same protein source. Data are means ± SD.

Myofibrillar FSR in the non-exercise rested leg (fed only) was unchanged in response to ingestion of soy protein in both the S20 and S40 group, but increased in response to whey in both the W20 and W40 group (Figure 5). As such, MPS was significantly greater following whey vs. soy protein with ingestion of both 20 g and 40 g protein (both *P* < 0.005). In the exercised condition, myofibrillar FSR was no different for S20, but increased for S40 when compared to the 0 g group. However, the response of MPS to soy was less than that of whey at both protein doses in the exercised condition (both *P* < 0.001; Figure 5).

## Discussion

In the present study, we show that ingestion of 20 g (S20) and 40 g (S40) of soy protein isolate does not stimulate increased rates of MPS under resting conditions in the elderly. However, when combined with the potent anabolic stimulus of resistance exercise, 40 g but not 20 g, of soy protein isolate has a modest effect on increasing post-exercise rates of MPS when compared to a group who performed resistance exercise without subsequent protein intake (Figure 5). These data are in contrast to what was observed following equivalent doses of whey protein, as 20 g (W20) effectively stimulated MPS at rest, while both 20 g and 40 g (W40) increased post-exercise MPS in a stepwise manner (i.e. 40 g > 20 g) [6]. Thus, when comparing our current findings on soy protein with our previous work examining graded doses of whey protein [6], soy appears less effective than whey protein at promoting increases in MPS in the elderly (Figure 5). Further, our results confirm that the elderly benefit from significantly greater doses of protein after exercise [6,12] than do the young, who we have shown mount a maximal MPS response with ingestion of ~20 g protein [5] or ~10 g EAA [2].

We have previously reported that soy protein is less effective than whey [9] and bovine milk protein [8] at increasing rates of post-exercise muscle protein synthesis in young subjects. Whey protein appears superior in its ability to stimulate muscle protein synthesis not only when compared to soy, but also when compared to other dairy protein sources such as intact [7,9,10] or hydrolyzed [10] casein. The mechanism(s) underpinning differences in the capacity of these proteins to support increased rates of MPS has not been fully elucidated. Previous research in rats reported greater increases in the phosphorylation status of mTOR(Ser 2448) and p70S6k (Thr 389), critical proteins involved in regulating translation initiation of protein synthesis, following whey compared with soy protein intake after endurance exercise [30]. Other important factors may relate to important differences in the leucine content of the respective proteins (~12% in whey and ~8% in soy) [16], and/or to differences in their digestion/absorption kinetics and the subsequent aminoacidemia [17,18,31]. For example, protein digestibility has been established as an important factor regulating whole-body protein synthesis and breakdown [17,18]; rapidly digested proteins have been shown to elicit a large increase in whole-body protein synthesis, whereas ‘slow’ proteins reduce rates of whole-body proteolysis [17,18,32]. More recent work has extended these findings at the whole-body level by showing that a fast protein, such as whey, also stimulates greater rates of skeletal muscle protein synthesis than does a slow protein, such as casein, both in both young and elderly subjects [7,9,10]. However, although whey and soy are relatively rapidly digested dietary proteins [19,33], previous studies have demonstrated that the amino acids from soy are partitioned for use within the body by more rapidly turning-over gut (i.e. splanchnic) proteins, and are converted to urea to a greater extent than amino acids from dairy based proteins which are partitioned to the periphery for use by skeletal muscle tissue [19,34].

In the present study, we observed protein source-dependent differences in rates of leucine oxidation (Figure 4). When expressed relative to lean body mass, rates of leucine oxidation were significantly greater for S20 than W20 (Figure 4). The higher rates of leucine oxidation in S20 vs. W20 suggest that a greater proportion of the amino acids from soy protein were diverted towards oxidation, and were thus unavailable as substrate for protein synthesis. Overall, although they are considered to be equivalent high quality proteins from the perspective of the truncated PDCAAS scoring system [16], there are clearly important differences in the capacity of soy and whey protein to stimulate MPS and promote anabolism. This point is of particular importance to the elderly in whom preserving skeletal muscle mass is of importance. Previous work showing that nitrogen balance is attainable with long-term diets containing moderate amounts of soy [35] would appear to be incongruent with our data; however, these data [35] are confounded by weight loss in a number of the subjects and due to the age of subjects in this study not being entirely comparable. Our data would, in contrast to previous conclusions regarding the adequacy of soy protein [35-37], suggest that long-term consumption of soy protein may not attenuate sarcopenic muscle loss.

The mechanisms underpinning the ‘anabolic resistance’ of elderly muscle to nutrient provision are not entirely clear. Given the results of the current study, and previous studies demonstrating that MPS responds favorably to higher doses of protein in the elderly [6,12] as compared to the young [5], it appears that the muscle of older persons has a higher anabolic aminoacidemic ‘threshold’ [6,9,38] that can be surpassed by ingesting either greater quantities of protein/amino acids or possibly greater leucine [13]. The greater rates of MPS observed with equivalent doses of whey as compared to soy protein suggest that protein source is an important factor in reaching and surpassing the anabolic threshold (Figure 5). The branched chain amino acid leucine has been shown to be a key activator of muscle protein synthesis through its ability to regulate mRNA translation initiation through the mTOR signaling pathway [39,40]. For example, Katsanos and colleagues [13] reported that while 6.7 g of EAA containing ~26% leucine failed to stimulate MPS in the elderly, increasing the leucine content to ~41% increased MPS in the elderly such that measured rates were not different from that seen in the young. Based on results from the present study, there were no protein source dependent differences in leucine area under the curve (AUC) at either the 20 g or 40 g dose (Figure 3), however, the temporal response of blood leucine was different following whey and soy at both protein doses (Figure 2) with the response of whey being greater in amplitude than that observed following soy. To overcome the confounding influence of amino acid composition when comparing different proteins, we recently manipulated the pattern of postprandial aminoacidemia using a bolus versus a pulsed feeding pattern with whey protein [41]. Despite equivalent leucine and EAA AUC (i.e., net exposure) the bolus feeding pattern and the associated rapid aminoacidemia stimulated greater rates of post-exercise MPS than pulse feeding, which elicited a moderate but sustained rise in aminoacidemia [41]. Further, supplementation of soy protein with the BCAA has been shown to increase the anabolic effect of this protein in both the elderly and clinical COPD patients [42]. Thus, the higher leucine content and more rapid leucinemia with whey as opposed to soy may in part explain the observed differences in resting and post-exercise MPS between the two proteins.

In summary, we report that soy protein isolate is relatively ineffective in its capacity to stimulate MPS in the elderly when compared to whey protein. The mechanisms underpinning the reduced anabolic effect of soy as compared to whey likely relate to its relatively lower leucine content (~12% in whey and ~8% in soy) [16] and reduced leucinemia as a result of subtle differences in digestion/absorption between soy and whey protein. It is unlikely these differences have a marked impact on protein nutrition in all but the elderly or clinical populations [42]. Differences in postprandial amino acid oxidation rates may also be important as lower doses of soy (S20) resulted in greater increases in leucine oxidation than equivalent doses of whey protein. Our results have implications for nutrient formulations designed to support increased muscle protein anabolism in the elderly and suggest that whey protein offers clear advantages to soy protein in its capacity to support both rested and post-exercise increases in MPS.

## Abbreviations

MPS, myofibrillar protein synthesis; S20, 20 g soy protein isolate; S40, 40 g soy protein isolate; SPPB, short physical-performance battery; W20, 20 g whey protein isolate; W40, 40 g whey protein isolate.

## Competing interests

YY, TACV, NAB, LB, MAT, and SMP declare that they have no competing interests.

## Authors’ contributions

YY and SMP designed the research; YY, TACV, NAB, MAT and SMP conducted the research; YY and SMP analyzed the data; YY, TACV, LB, and SMP wrote and edited the manuscript; SMP had primary responsibility for the final content. All authors read and approved the final content.

## Supplementary Material

Additional file 1 **Table S1.** Participants’ dietary intake. (PDF 129 kb) Click here for file

Additional file 2 **Table S2.** Amino acid profiles of the whey (W20, W40) and soy (S20, S40) protein drinks . Click here for file
