# Supplementary material for: Myofibrillar protein synthesis following ingestion of soy protein isolate at rest and after resistance exercise in elderly men
Source: Nutr Metab (Lond). 2012 Jun 14;9:57. doi: 10.1186/1743-7075-9-57 (PMC3478988; doi:10.1186/1743-7075-9-57)
Supplement: Additional file 1 — Table S1. Participants’ dietary intake. (PDF 129 kb) [file 1743-7075-9-57-S1.pdf]

Supplementary Table 1. Participants' dietary intake

	0 g (n = 10)	W20 (n = 10)	W40 (n = 10)	S20 (n = 10)	S40 (n = 10)
Total energy (kcal)	2327	2288	2388	2182	2192
% Target energy	100	98	99	100	100
Protein ($g \cdot kg^{-1} d^{-1}$)	1.03	1.04	1.03	1.03	1.04
% Carbohydrate	59	61	58	59	59
% Protein	14	15	14	15	15
% Fat	27	24	28	26	26

Dietary intake calculated over 2 days immediately prior to the infusion trial.

Values are means ± SD.
