# Supplementary material for: Myofibrillar protein synthesis following ingestion of soy protein isolate at rest and after resistance exercise in elderly men
Source: Nutr Metab (Lond). 2012 Jun 14;9:57. doi: 10.1186/1743-7075-9-57 (PMC3478988; doi:10.1186/1743-7075-9-57)
Supplement: Additional file 2 — Table S2. Amino acid profiles of the whey (W20, W40) and soy (S20, S40) protein drinks . [file 1743-7075-9-57-S2.pdf]

Supplementary Table 2. Amino acid profiles of the whey and soy protein drinks

Amino Acid (g)	**0 g (n = 10)**	**W20 (n = 10)**	**W40 (n = 10)**	**S20 (n = 10)**	**S40 (n = 10)**
Isoleucine	-	1.4	2.8	1.0	2.0
Leucine	-	2.0	4.0	1.6	3.2
Lysine	-	1.8	3.6	1.3	2.6
Methionine	-	0.4	0.8	0.3	0.6
Threonine	-	1.4	2.8	0.7	1.4
Phenylalanine	-	0.6	1.2	1.0	2.0
Tryptophan	-	0.4	0.8	0.2	0.4
Valine	-	1.2	2.4	1.0	2.0
Histidine	-	0.4	0.8	0.5	1.0
Alanine	-	1.0	2.0	0.9	1.8
Arginine	-	0.4	0.8	1.5	3.0
Aspartic acid	-	2.2	4.4	2.3	4.6
Cystine	-	0.4	0.8	0.2	0.4
Glutamic acid	-	3.4	6.8	3.8	7.6
Glycine	-	0.4	0.8	0.8	1.6
Proline	-	1.2	2.4	1.0	2.0
Serine	-	0.8	1.6	1.1	2.2
Tyrosine	-	0.6	1.2	0.8	1.6
Total EAA	-	9.2	18.4	7.1	14.2
Total BCAA	-	5.2	10.4	3.9	7.8
Total AA	-	20.0	40.0	20.0	40
